# Characterization of Epistatic Interaction of QTLs *LH8* and *EH3* Controlling Heading Date in Rice

**DOI:** 10.1038/srep04263

**Published:** 2014-03-03

**Authors:** Jingbin Chen, Xiaoyan Li, Cheng Cheng, Yahuan Wang, Mao Qin, Haitao Zhu, Ruizhen Zeng, Xuelin Fu, Ziqiang Liu, Guiquan Zhang

**Affiliations:** 1 State Key Laboratory for Conservation and Utilization of Subtropical Agro-Bioresources, Guangdong Key Laboratory of Plant Molecular Breeding, College of Agriculture, South China Agricultural University, Guangzhou 510642, China; 2 Institute of Vegetable Crops, Jiangsu Academy of Agricultural Sciences, Nanjing 210014, China; 3 College of Agronomy, Northwest Agriculture & Forestry University, Yangling, 712100, China

## Abstract

Heading date is a critical trait for adaptation of rice to different cultivation areas and cropping seasons. We evaluated the heading dates of 1,123 chromosome segments substitution lines (CSSLs) in the genetic background of an elite rice variety Huajingxian74 (HJX74). A CSSL with the substituted segments from Zihui100 exhibited late heading under both natural long-day (NLD) and natural short-day (NSD) conditions, and the late heading phenotype was controlled by two novel epistatic loci on chromosome 8 and chromosome 3, respectively, termed *LH8* and *EH3*. The function of *EH3* was dependent on the *LH8* genotype through epistatic interaction between *EH3*^Zihui100^ and *LH8*^Zihui100^ alleles. Genetic and molecular characterization revealed *LH8* encodes a CCAAT-box-binding transcription factor with *Heading date1* (*Hd1*)-binding activity and may delay flowering by repressing the expression of *Early heading date1* (*Ehd1*). Our work provides a solid foundation for further study on gene interaction in heading date and has application in breeding rice with greater adaptability.

Rice is a short-day (SD) plant, and is becoming an increasingly important model monocot plant for molecular biological study on agronomic important traits such as heading date. Heading date, flowering time in cereal crops, is a critical agronomical determinant for adaptation to specific cropping locations and growing seasons for current varieties of cultivated rice and is an important consideration for rice breeders. The molecular and developmental determinants of heading date have thus particularly important genetic targets for domestication and for breeding new varieties of rice.

The timing of flower initiation is controlled by both environmental and internal signals^1^,^2^. By taking advantage of pioneering works in long-day (LD) plant *Arabidopsis*, many molecular components of rice flowering process initiated from photoperiod and light signals have been identified through natural variations, quantitative trait locus (QTL) analyses and by screening mutant populations (as summarized and reviewed in^3^). Two independent flowering pathways, the conserved *Hd1*-dependent, and unique *Ehd1*-dependent pathways, control heading time in rice^4^,^5^,^6^,^7^. The two pathways function redundantly under SD conditions but antagonistically under LD conditions^8^. *Hd1*, a rice ortholog of *Arabidopsis CONSTANS* (*CO*) that is regulated by *OsGI* (a rice ortholog of *Arabidopsis GIGANTEA*), activates flowering under SD conditions and represses it strongly under LD conditions through regulating the expression of *Heading date3a* (*Hd3a*), a rice ortholog of *Arabidopsis* florigen *FLOWERING LOCUS T* (*FT*)^4^,^9^. *Ehd1* encodes a B-type response regulator that is highly conserved in cultivated rice, but has no homolog in *Arabidopsis*^6^. *Ehd1* can promote flowering by activating the expression of *Hd3a* and *RICE FLOWERING LOCUS T1* (*RFT1*), the closest paralog of *Hd3a*^6^,^7^,^10^. The expression of *Ehd1* is promoted by a number of positive regulators, including *RID1* (also known as *OsID1* and *Ehd2*)^11^,^12^,^13^, *Early heading date3* (*Ehd3*)^14^, *Early heading date4* (*Ehd4*)^15^, *OsMADS51*^16^ and *OsMADS50* (also known as *DTH3*)^17^,^18^,^19^. On the other hand, several factors repress the expression of *Ehd1*, thereby delaying flowering, such as *Ghd7*^20^, *DTH8* (also known as *Ghd8* and *LHD1*)^21^,^22^,^23^, *SE5*^24^, *OsCOL4*^25^ and *OsLFL1*^26^,^27^. Therefore, *Ehd1* serves as a critical convergence point of regulation by multiple signaling pathways. The flowering signals are collected from *Hd1* and *Ehd1*, and are transducted to a pair of florigen genes, *Hd3a* and *RFT1*, promoting heading under SD and LD conditions, respectively^7^,^10^. After production in leaves, these florigens move to the shoot apical meristem and promote floral transition by activating the expression of *OsMADS14* and *OsMADS15*^7^. Although these studies have revealed much insight into the flowering control of rice, the underlying molecular mechanisms, especially the genetic interactions among these factors, are still not well understood.

The advantages of CSSLs in precise and detailed phenotype evaluation, minor QTL detection and validation of gene-gene and gene-environment interactions facilitated both genetic studies and rice improvement^28^,^29^,^30^. Therefore, we had constructed a library of 1,123 CSSLs in rice using HJX74, an elite *indica* variety from south China, as recipient and 24 accessions, including 14 *indica* and 10 *japonica*, collected worldwide as donors to understand the molecular mechanism of agronomic traits including heading date^31^,^32^. Each CSSL contains a very small number of well-characterized chromosome segments from one of the 24 donor varieties.

In this study, we found a CSSL with the substituted segments from Zihui100 exhibited late heading under both NLD and NSD conditions, and the late heading phenotype was controlled by two novel epistatic loci on chromosome 8 and chromosome 3, respectively, termed *LH8* and *EH3*. Map-based cloning of *LH8* revealed it encodes a CCAAT-box-binding transcription factor and is allelic to *DTH8*. Molecular function of *LH8* was further studied by expression analysis and yeast-two-hybrid experiment.

## Results

### Identification of a CSSL with late heading

**Figure 1:**
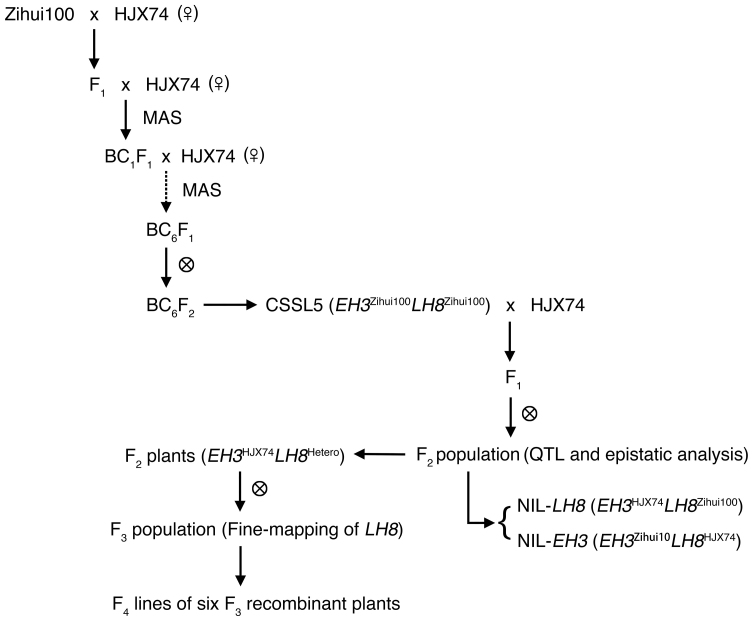
Flowchart of the development of CSSLs and QTL analysis. MAS: marker-assisted selection.

**Figure 2:**
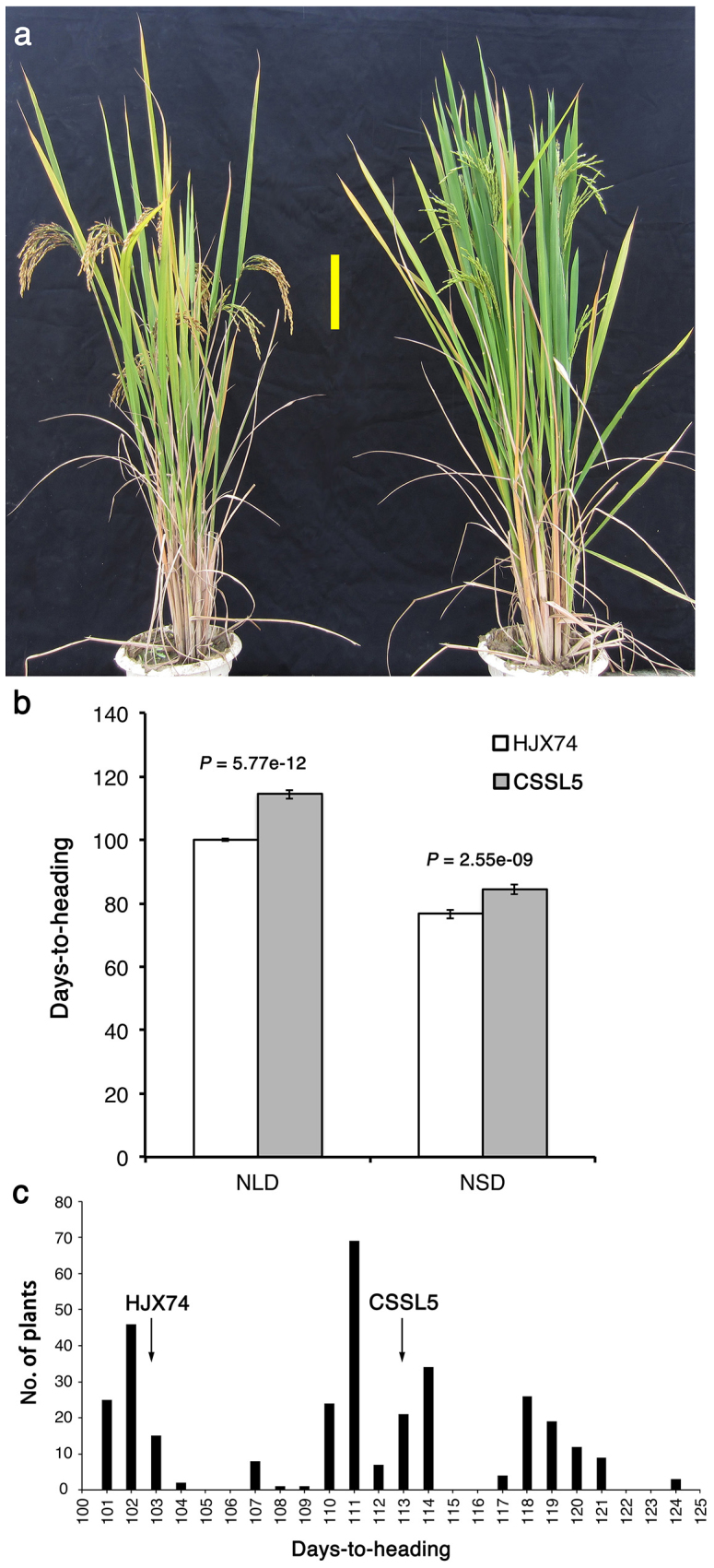
The heading dates of HJX74, CSSL5 and their F_2_ population. (a). Phenotypes of HJX74 (left) and CSSL5 (right). Yellow bar = 15 cm. (b). Days-to-heading in HJX74 and CSSL5 in different photoperiodic conditions. NLD, natural long-day conditions; NSD, natural short-day conditions. Each column represents mean ± s.d. (n = 30). (c). Frenquency distributions of days-to-heading in an F_2_ population from HJX74 x CSSL5 under NLD conditions. Black arrows indicate the mean DTH of HJX74 and CSSL5 (n = 30), respectively.

The procedure for the development of the CSSLs was summarized in Fig. 1. HJX74 (as the female parent) was crossed with one of the 24 donors (Zihui100 was taken as an example), and the F_1_ plants were backcrossed with HJX74 to develop the BC_1_F_1_ generation. Polymorphic SSR markers were used in the selection of the donor chromosomal segments. Using the same method, BC_6_F_1_ plants were obtained, and were self-crossed to produce BC_6_F_2_ lines in which the majority of genomic regions were homozygous for HJX74 alleles. Totally, 1,123 such CSSLs in the HJX74 genetic background were developed^31^,^32^. To further understand how flowering in rice was controlled, we evaluated the heading date of the 1,123 CSSLs under natural growth conditions. A CSSL, CSSL5, with substituted segments from Zihui100 exhibited late heading compared with the recipient HJX74 under both NLD (114.4 ± 1.4 d for CSSL5, 100.1 ± 0.5 d for HJX74) and NSD conditions (84.5 ± 1.6 d for CSSL5, 76.7 ± 1.4 d for HJX74) in 2009 (Fig. 2a and 2b).

To analyze the genetic basis for the late heading of CSSL5, we crossed CSSL5 with the recipient HJX74 (Fig. 1). 73 out of 326 F_2_ plants showed heading date later than that of CSSL5 (113.0 ± 1.3 d) under NLD conditions in 2010, and the whole population exhibited trimodal distribution of heading date fitting 4:9:3 segration ratio (*χ^2^* = 4.67 < *χ^2^*_0.05,2_ = 5.99) (Fig. 2c). These results indicate that the late heading phenotype was controlled by two genes with negative epistasis.

### Confirmation of the two genes by resequencing

**Figure 3:**
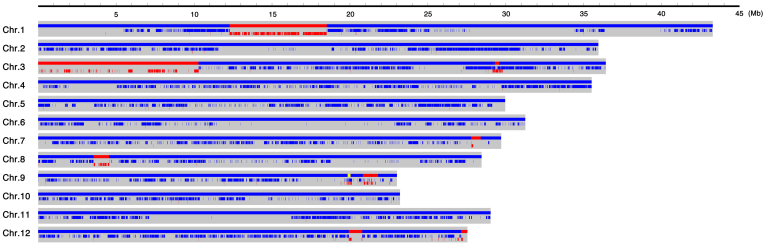
Physical map of CSSL5 based on the whole-genome resequencing data. Red areas indicated the substituted chromosome segments from Zihui100, while the blue areas indicated chromosome regions of HJX74. The tiny yellow area on chromosome 9 indicated a heterozygous segment between HJX74 and Zihui100.

To delimit the heading date genes on the substituted segments, CSSL5 and HJX74 were high-throughput genotyped by whole-genome resequencing, and an ultrahigh-quality physical map was constructed (Fig. 3). 11 substituted segments with a total length of 20.64 Mb derived from Zihui100 were found to be distributed over 6 chromosomes (Fig. 3 and Supplementary Table S1 online). Molecular markers were subsequently designed on the substituted segments, and were used to analyze their linkage with heading date phenotype of the F_2_ population. Two QTLs were identified on chromosome 3 and 8, respectively, designated as *EH3* and *LH8*, respectively. *EH3* was mapped in the 750 kb genomic region between the Id32 and Id33 markers and had the contributions to phenotypic variation by 14.0%, the allele from Zihui100 could shorten the heading date by 2.32d and the dominant effect was −3.24. *LH8* was mapped in the 460 kb genomic region between the Id83 and Id82 markers and had the contributions to phenotypic variation by 65.3%. The allele from Zihui100 could delay the heading date by 6.62d and the dominant effect was 3.44 (Table 1).

### Epistatic interaction between *EH3* and *LH8*

**Figure 4:**
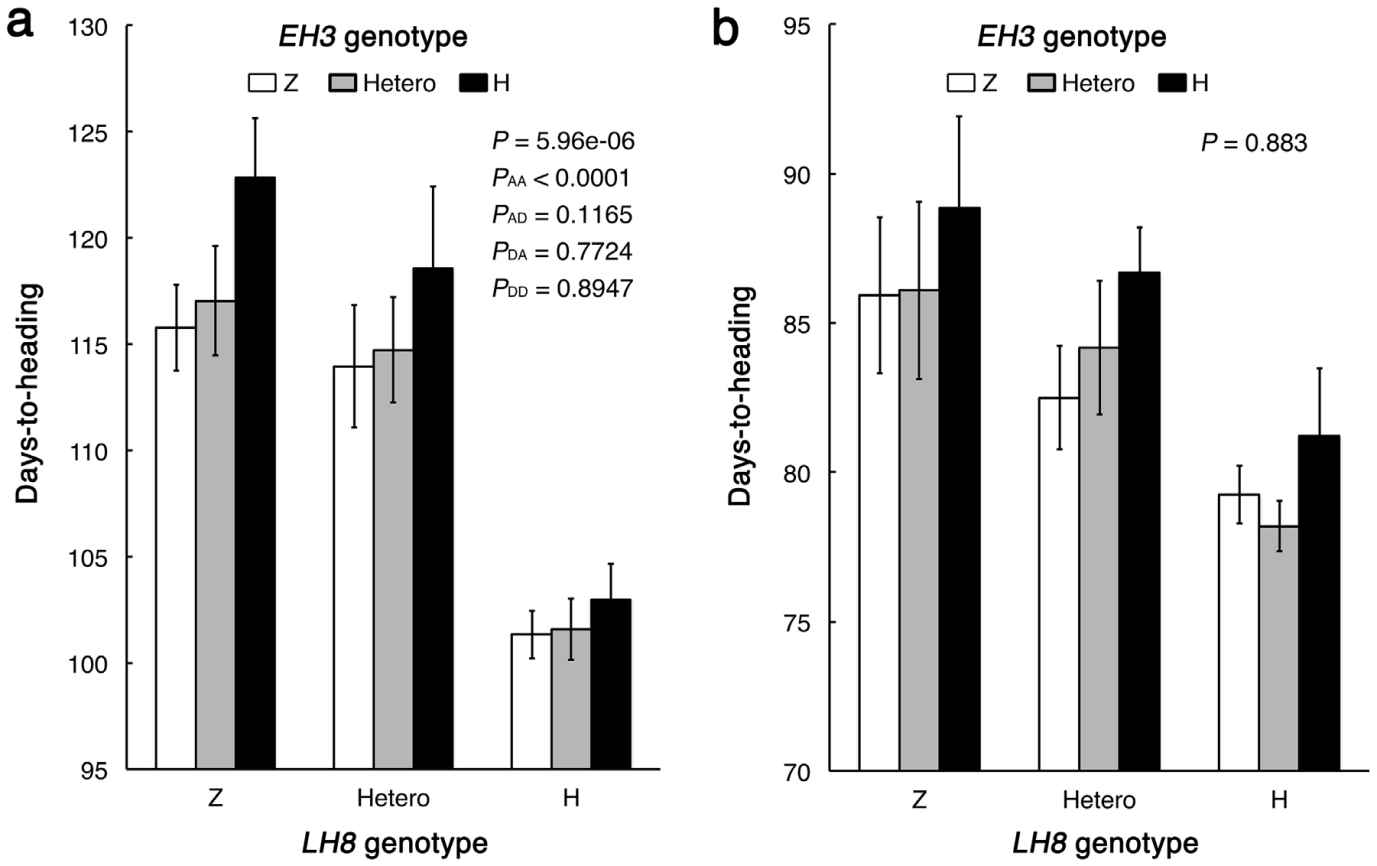
Epistatic interaction between *LH8* and *EH3*. Differences in days-to-heading for nine genotype classes for combinations of *LH8* and *EH3* under NLD (a) and NSD (b) conditions in the F_2_ populations. Genotypes were determined using the closely linked markers Id83-Id82 (*LH8*) and Id32-Id33 (*EH3*). Z, Hetero and H indicate homozygous for Zihui100 allele, heterozygous and homozygous for the HJX74 allele, respectively. *P*_AA_, *P*_AD_, *P*_DA_ and *P*_DD_ are significant probalities for AA, AD, DA and DD interactions between *LH8* and *EH3*, respectively.

To examine the genetic interactions between *EH3* and *LH8*, we scored heading dates in the F_2_ population under NLD and NSD conditions in 2011 (Fig. 4). The F_2_ plants were classified into nine genotype classes based on the *EH3* and *LH8* alleles, and we compared the average heading date among the nine classes. Under NLD conditions, two-way ANOVA revealed the existence of digenic interaction between *EH3* and *LH8* (*P* = 5.96e-06). Orthogonal contrast test showed that AA (Additive by additive), but not AD (Additive by dominance), DA (Dominance by additive) or DD (Dominance by dominance) type of interaction was significant in the interaction of *EH3* and *LH8* (*P* < 0.0001). The effect of the Zihui100 allele at *LH8* (i.e., increased days-to-heading (DTH)) was regardless of the allele status of *EH3*. In comparison, the effect of the Zihui100 allele at *EH3* (i.e., shortened DTH) was observed in two genotype classes, homozygous for the Zihui100 allele at the *LH8* locus and heterozygous, but not in the class homozygous for the HJX74 allele at the *LH8* locus (Fig. 4a). Under NSD conditions, the Zihui100 allele at *LH8* was also observed to delay heading regardless of the allele status of *EH3*. However, the two-way ANOVA suggested the digenic interaction between *EH3* and *LH8* was not significant under NSD conditions (*P* = 0.883) (Fig. 4b).

### Map-based cloning of *LH8*

**Figure 5:**
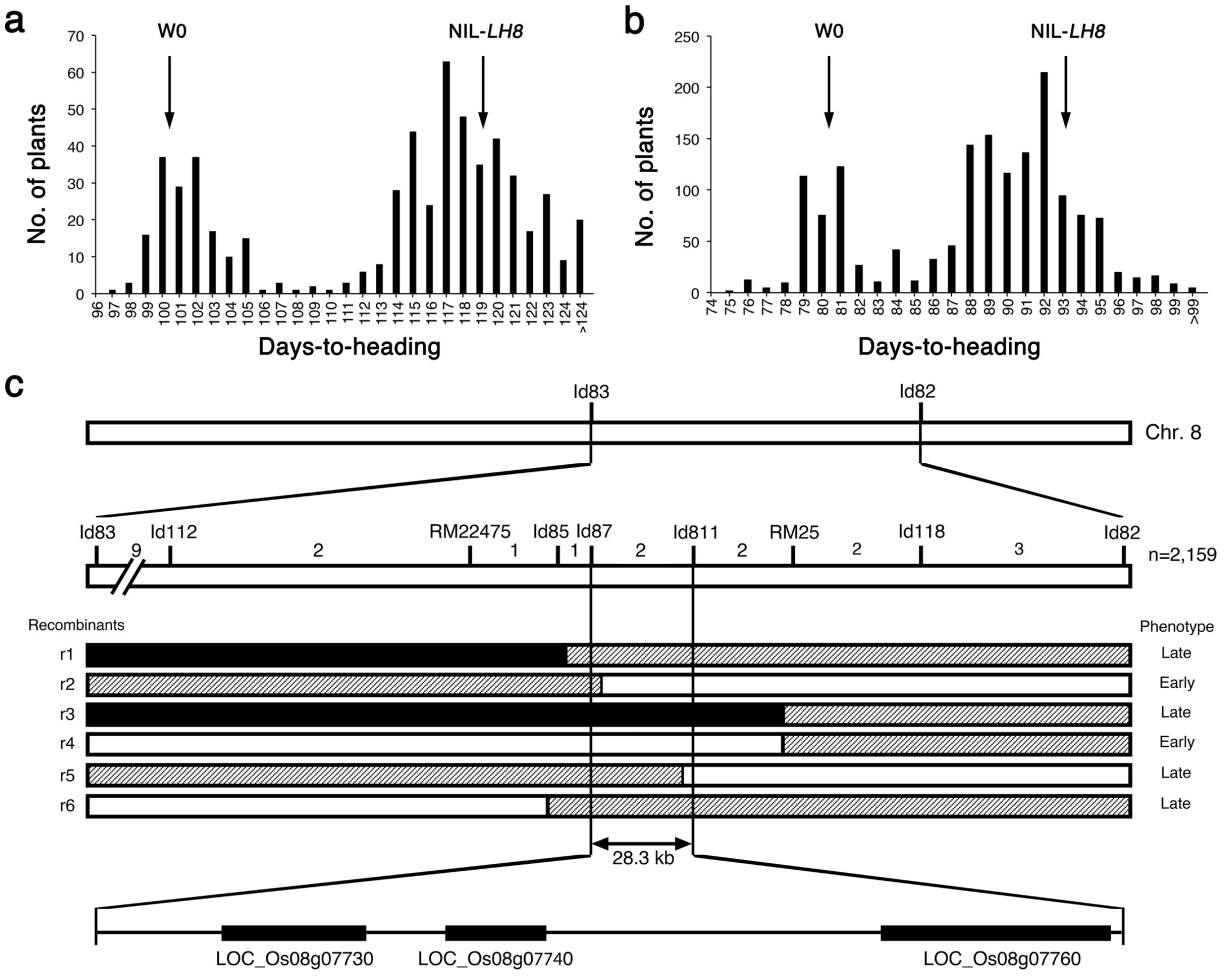
Map-based cloning of the *LH8* gene in rice. (a and b). Frenquency distributions of days-to-heading in an F_3_ population from HJX74 x CSSL5 under NLD (a) and NSD (b) conditions. Black arrows indicate the mean DTH of HJX74 and NIL-*LH8* (n = 30), respectively. (c). High-resolution mapping of *LH8*. The genetic map of *LH8* was based on recombinant events among 2,159 F_3_ plants from HJX74 x CSSL5. The number of recombinants between adjacent markers is shown above the bar. White, shadow and black regions shown in the recombinants indicate homozygous regions for HJX74 allele, heterozygous and homozygous regions for the Zihui100 allele, respectively.

**Figure 6:**
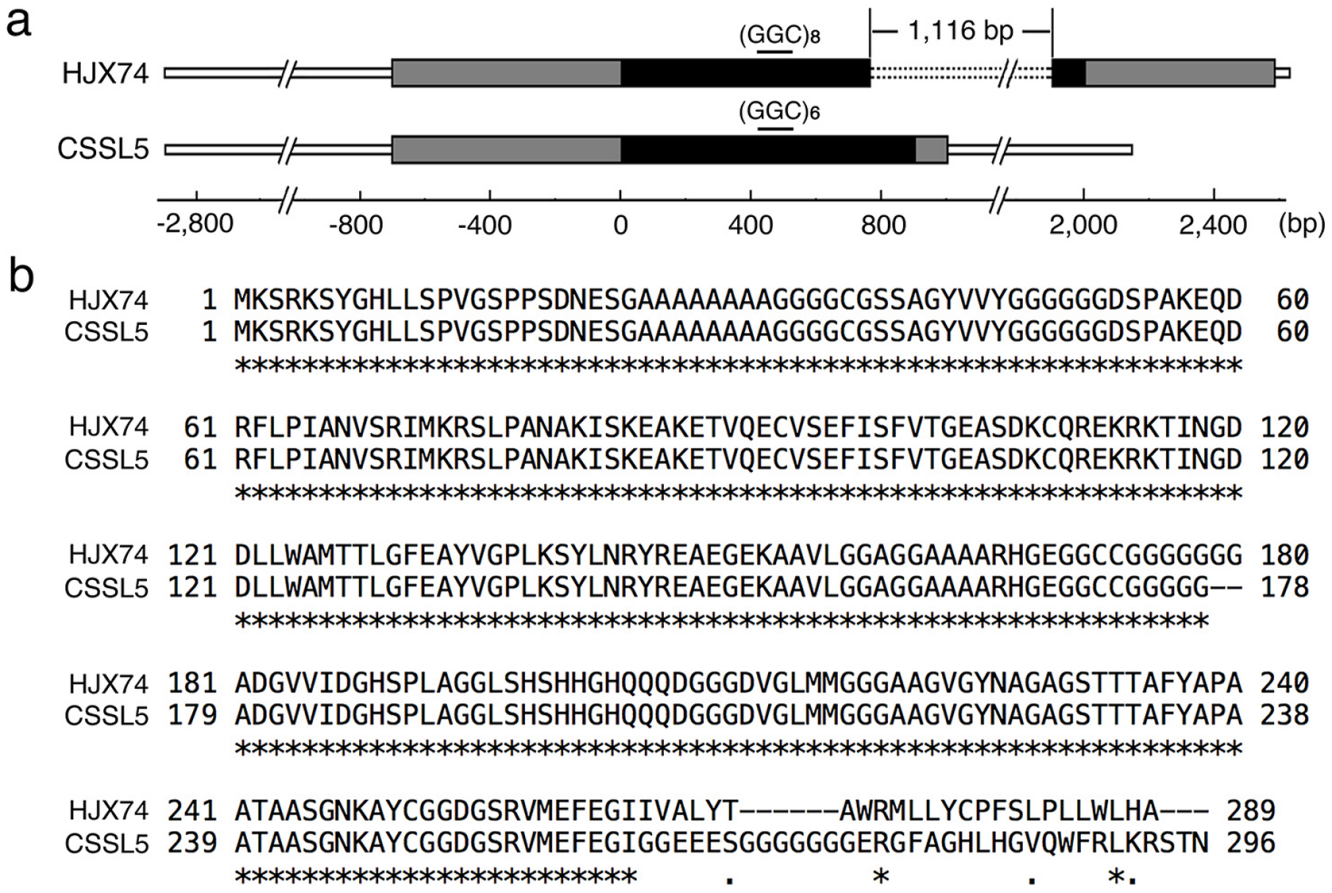
Sequence comparision of *LH8* from HJX74 and CSSL5. (a). Structure of *LH8* in HJX74 and CSSL5. Black and gray regions represent the ORF and UTR regions of *LH8*, respectively. The dotted line represents the 1,116 bp deletion in HJX74. (b). Protein alignment of LH8 from the predicted protein of HJX74 and CSSL5 using the EMBL software ClustalW2 multiple sequence alignment tool.

To fine map the *LH8* gene, we selected F_2_ plants with homozygous *EH3* allele from HJX74 and heterozygous *LH8* allele from Zihui100 to generate F_3_ population (Fig. 1). F_3_ population showed bimodal distribution for DTH fitting 3:1 segregation ratio under both NLD and NSD conditions in 2012 (*χ^2^* = 0.04 < *χ^2^*_0.05,1_ = 3.84 and *χ^2^* = 0.03 < *χ^2^*_0.05,1_ = 3.84 for NLD and NSD, respectively), indicating the Zihui100 allele at *LH8* increased DTH in a dominant manner (Fig. 5a and 5b). 7 new markers were developed in the marker interval of Id83-Id82, and were used to analyze a total of 2,159 F_3_ plants. 6 recombinants were identified between markers RM22475 and RM25 (Fig. 5c). The self-pollinated progeny (F_4_ lines) of those 6 plants were used to determine the genotypes of *LH8* (Fig. 1). The recombinants r2 and r5 restricted *LH8* to the 28.3 kb genomic region between the Id87 and Id811 markers, including 3 putative genes, a transferase family protein (LOC_Os08g07730), a SERK-family receptor-like protein kinase (LOC_Os08g07760) and a CCAAT-box-binding transcription factor (LOC_Os08g07740) which had been reported to function in heading date control in rice (Fig. 5c). The sequencing of *LH8* genomic regions from CSSL5 and HJX74 revealed two GGC insertion and a 1,116-bp deletion in LOC_Os08g07740 of HJX74 (Fig. 6a), resulting in a 2-glycine insertion in the middle and a big alternation in the C-terminal region of the translated protein, respectively (Fig. 6b). These results suggest that LOC_Os08g07740 corresponds to *LH8* controlling the late heading in CSSL5.

### Characterization of *LH8* in rice

**Figure 7:**
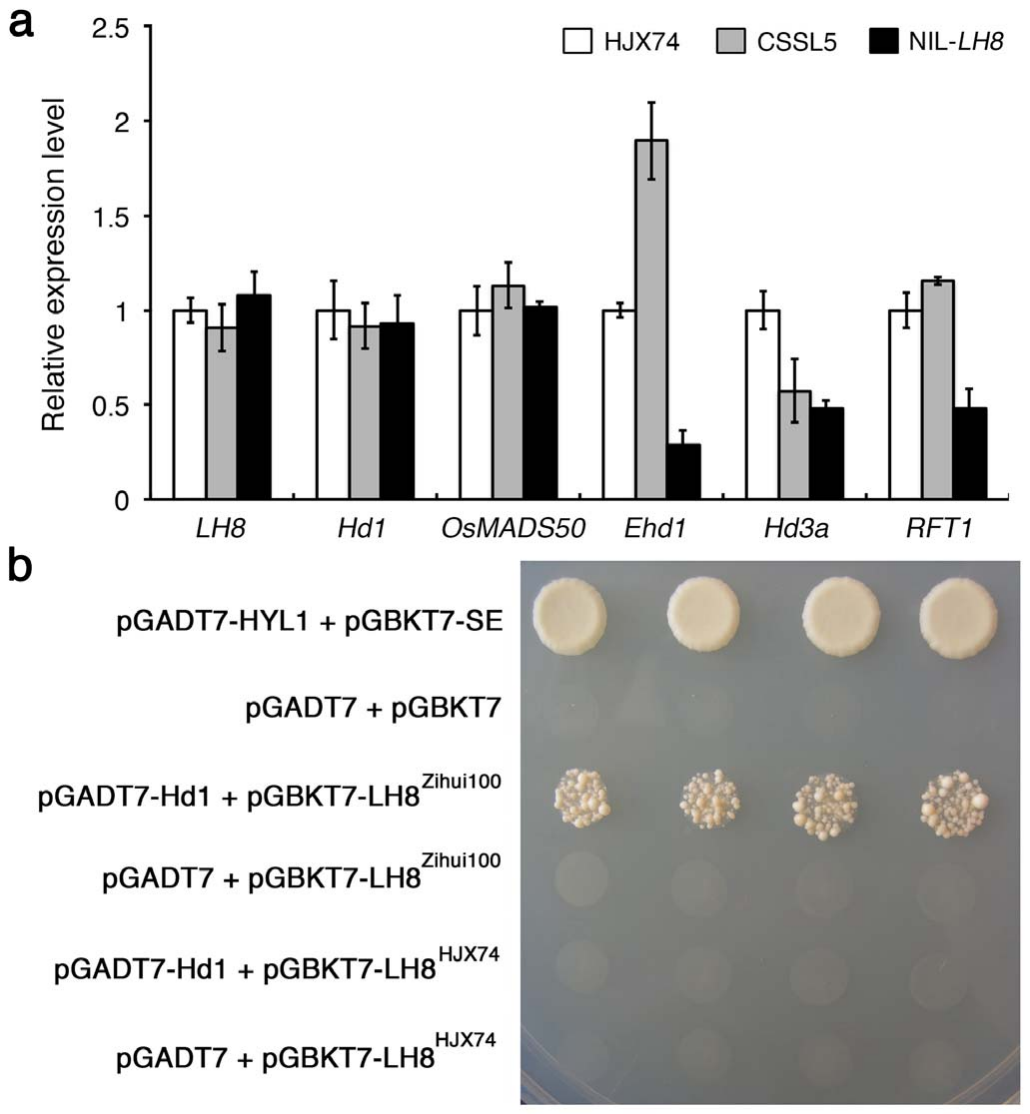
Characterization of the *LH8* gene in rice. (a). Expression of *LH8*, *Hd1*, *OsMADS50*, *Ehd1*, *Hd3a* and *RFT1* in HJX74 (white pillars), CSSL5 (gray pillars) and NIL-LH8 (black pillars) at dawn (for *LH8*, *OsMADS50*, *Ehd1*, *Hd3a* and *RFT1*) and dusk (for *Hd1*) under NSD conditions. The analysis was performed in two independent experiments. The mean values of the relative expression levels of genes in CSSL5 and NIL-*LH8* compared with HJX74 are shown by cloumns, and standard deviations are indicated by the error bars. (b). Interaction of LH8 and Hd1 by yeast-two-hybrid assay. The yeast cells were grown on SD/-Leu/-Trp/-His/-Ade medium. The interaction between SE and HYL1 was served as positive control, while the combination of empty prey pGADT7, and that of the empty bait vector pGBKT7 with/without LH8 from Zihui100 were served as negative controls.

To examine whether the sequence mutations can alter the expression of *LH8*, we examined the expression levels of *LH8* in CSSL5, HJX74 and NIL-*LH8* (a line selected from *LH8* F_2_ segragating population with homozygous *EH3* allele from HJX74 and homozygous *LH8* allele from Zihui100) under NSD conditions in 2013. No significant difference in the *LH8* expression levels could be detected among the three materials, suggesting that late heading did not result from the *LH8* transcription. The expression levels of other heading date genes were also analyzed. For *Hd1* and *OsMADS50*, no difference could be detected among the three materials under NSD conditions. The expression levels of *Ehd1*, *Hd3a* and *RTF1* were less in NIL-*LH8* than those in HJX74 under NSD conditions, indicating that *LH8* may delay heading through suppressing the expressions of these floral transition activators. In contrast, the expression levels of *Ehd1* and *RTF1* were higher in CSSL5 than those in HJX74. The different expression levels of *Ehd1* and *RFT1* between CSSL5 and NIL-*LH8* may result from the function of different *EH3* alleles (Fig. 7a).

To study the involvement of the 2-glycine indel and the C-terminal region of the LH8 protein in its function, we tested the interaction between LH8 and Hd1 by yeast-two-hybrid method. Yeast cells that coexpressed the LH8^Zihui100^ bait and Hd1 prey fusion proteins were able to grow on media lacking histidine and adenine, indicating that these proteins can bind each other in yeast cells to promote the expression of the HIS3 and ADE2 reporter genes. However, the similar interaction could not be detected between LH8^HJX74^ and Hd1, indicating the 2-glycine indel and the C-terminal region of LH8 might be important for its interaction with Hd1 (Fig. 7b).

## Discussion

**Figure 8:**
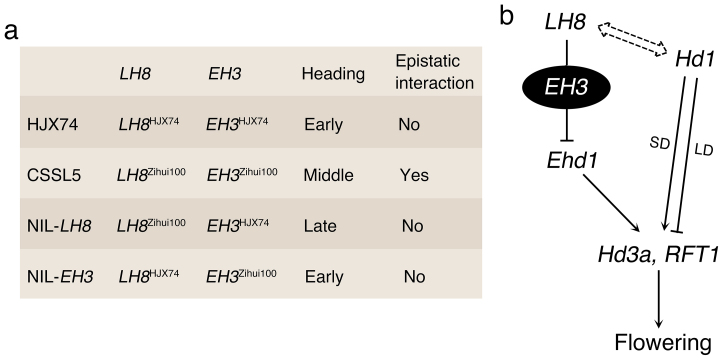
Schematic representation of the *LH8* and *EH3* mediated flowering pathway under natural growth conditions. (a). Epistatic model of rice heading controlled by *LH8* and *EH3*. (b). A proposed model for the flowering pathway controlled by *LH8* and *EH3* in rice under natural growth conditions. *LH8* could down-regulate *Ehd1*, which could act on *Hd3a* and *RFT1*, thus delaying flowering in rice under natural growth conditions. *EH3* might function as a modifier of *LH8* to shorten heading date only when specific *LH8* background was present. The interaction between LH8 and Hd1 depended on different alleles presented in different genetic backgrounds, possibly resulting in varied functions in flowering control.

Until now, a total of 734 rice heading date QTLs have been reported (http://www.gramene.org/qtl), and some of them have been molecularly characterized^3^. Besides understanding the biological function of single gene/QTL, clarification of genetic interactions among these genes/QTLs is also important. Actually, several examples of epistatic interactions among heading date genes/QTLs have been reported^33^,^34^,^35^,^36^,^37^. By eliminating genetic background noises, CSSLs offer a practical solution to bridge the huge gap of knowledge between the genotype and phenotype, and therefore, are especially useful in studying complicated traits including heading date. In this study, a novel epistatic interaction between *LH8* and *EH3* involved in flowering control in rice was detected. Under NLD conditions and HJX74 genetic background, in plants homozygous for the *LH8*^HJX74^ allele, difference in DTH between *EH3*^Zihui100^ and *EH3*^HJX74^ alleles was small (about 1 day), as shown in HJX74 and NIL-*EH3* (a line selected from *LH8* F_2_ segragating population with homozygous *LH8* allele from HJX74 and homozygous *EH3* allele from Zihui100) (Fig. 8a). A large effect of *EH3*^Zihui100^ allele on heading promotion (about 7 days) occurred with the *LH8*^Zihui100^ allele, indicating *EH3*^Zihui100^ allele could function to shorten heading date only when *LH8*^Zihui100^ allele was concurrently present, and therefore, the function of *EH3* was dependent on the *LH8* genotype through epistatic interaction between *EH3*^Zihui100^ and *LH8*^Zihui100^ alleles (Fig. 8a). This result suggests that *EH3* might function as a modifier of *LH8*. Genetic interaction between *LH8* and *EH3* suggested that QTL pyramiding would be an effective method for the development of varieties with desirable heading dates under different growth conditions.

To facilitate the identification of the location of *LH8* and *EH3*, the high-throughput genotyping technology was exploited. The recipient HJX74 and CSSL5 were resequenced at the sequencing depth of 66- and 1-fold, respectively. Besides a large substituted segment from the short arm end to RM282 on chromosome 3 which was detected through traditional marker-assisted selection during CSSLs construction^31^, 10 additional substituted segments were identified distributing over 6 chromosomes, including some small introgressed segments that might be difficult to be detected by SSR markers (see Supplementary Table S1 online). Indeed, *LH8* was found on untargeted chromosomal region on chromosome 8. These results showed that the high-throughput genotyping method by resequencing has a higher accuracy than marker-assisted selection. Owning to the deep sequencing coverage of the recipient HJX74, resequencing the CSSL5 with genome coverage as low as 1-fold was enough to rapidly validate and delimit the QTL, suggesting the efficiency of this mapping-by-sequencing method in discovering the genes responsible for quantitative trait loci. Our results presented a good example of combining the advanced material with uniform genetic background and high-throughput resequencing technology in economically accelerating gene identification.

The sequencing of *LH8* genomic regions from CSSL5 and HJX74 revealed two GGC insertion and a 1,116-bp deletion in LOC_Os08g07740 of HJX74, resulting in a 2-glycine insertion in the middle and a big alternation in the C-terminal region of the translated protein, respectively (Fig. 6). Such sequence variations in the coding region of LOC_Os08g07740 between CSSL5 and HJX74 suggested that LOC_Os08g07740 might correspond to *LH8* controlling the late heading in CSSL5 and NIL-*LH8*. LOC_Os08g07740 encodes a CCAAT-box-binding transcription factor which had been reported to strongly inhibit floral transition with names of *DTH8*, *Ghd8* and *LHD1*^21^,^22^,^23^. Similar to *DTH8* and *Ghd8*, *LH8* increased plant height and grain number per panicle under NLD conditions (Fig. 2 and Supplementary Table S2 online), indicating *LH8* is allelic to *DTH8*, *Ghd8* and *LHD1*. Indeed, the two GGC insertion and one 1,116-bp deletion in the coding region of LOC_Os08g07740 of HJX74 had also been found in the ZS97 allele at *Ghd8*^22^. However, unlike *DTH8* and *LHD1* delaying flowering only under NLD/LD conditions^21^,^23^ and *Ghd8* functioning oppositely under SD and LD conditions^22^, *LH8* delays the heading date under both NSD and NLD conditions. These differences in function may come from different gene-background interactions or different growth conditions. Therefore, genes controlling important agronomic traits are necessary to be further studied in different genetic backgrounds and under different natural field conditions to give a full understanding of its effects and to offer practical instructions in rice breeding.

Expression analysis revealed the transcripts of several flowering promoting factors including *Ehd1*, *Hd3a* and *RFT1* were fewer in NIL-*LH8* than those in HJX74 under NSD conditions, suggesting *LH8* might delay flowering by down-regulating the expression of *Ehd1*, *Hd3a* and *RFT1* (Fig. 8b). The expressions levels of *Hd1* and *OsMADS50* showed no significant differences among HJX74, CSSL5 and NIL-*LH8* under NSD conditions, suggesting they might function upstream of *LH8* or parallel to *LH8*. Furthermore, no significant difference of *LH8* expression were found among HJX74, CSSL5 and NIL-*LH8* under NSD conditions, indicating the reason for the change of heading date was not caused by *LH8* expression, but by nucleotide changes of the coding sequence. The 6-bp and 1,116-bp indels in the coding region of *LH8* resulted in a big sequence alternation of the translated protein, especially in the C-terminal region.

The protein encoded by *LH8* is a putative HAP3 subunit of the HAP complex, which binds to the CCAAT box, a cis-acting element present in approximately 25% of eukaryotic gene promoters^38^,^39^. HAP proteins always form a HAP2/HAP3/HAP5 trimeric complex with DNA binding activity in mammals^40^,^41^. In *Arabidopsis*, CO could replace AtHAP2 in the HAP complex to form a trimetric complex, CO/AtHAP3/AtHAP5, as the CCT domain of CO and the DNA-binding domain of AtHAP2 are similar to each other^42^. In LD conditions, overexpression of *AtHAP2* or *AtHAP3* could delay flowering by impairing formation of the CO/AtHAP3/AtHAP5 complex, leading to decreased expression of *FT*^42^. As a homologue of CO, Hd1 may be able to replace OsHAP2 in the HAP complex in rice. Our yeast-two-hybrid results revealed that Hd1^HJX74^ could indeed interact with LH8^Zihui100^, but not with LH8^HJX74^, indicating the sequence difference, especially in the C-terminal region, between LH8^Zihui100^ and LH8^HJX74^ might be important for its interaction with Hd1^HJX74^, and therefore for the regulation of rice heading (Fig. 8b). The domains responsible for the interaction between LH8^HJX74^ and Hd1^HJX74^ were needed to be further investigated. However, the interaction between LH8^Zihui100^ and Hd1^Nip^ (Hd1 from Nipponbare) could not be detected (data not shown), in consistent with the previous study that effects of Ghd8 depend on the genetic background^22^. Consequently, whether the HAP complex could be formed or not was determined by the different subunits presented in different genetic backgrounds, possibly resulting in varied functions in flowering control.

Until now, 4 genes (*Ehd4*, *OsMADS50*, *OsDof12*, *OsPhyB*) on the short arm of chromosome 3 had been identified to be involved in flowering control in rice^15^,^17^,^18^,^19^,^43^,^44^. In our study, *EH3* was mapped in the 750 kb genomic region between the Id32 and Id33 markers with 123 putative ORFs inside, including *OsMADS50*. *OsMADS50*, an ortholog of *Arabidopsis* *SUPPRESSOR OF OVEREXPRSSION OF CONSTANS1* (*SOC1*), functions upstream of *Ehd1* to indirectly induce *RFT1* transcription^17^,^18^,^19^ and promote flowering. However, no epistatic interaction had been found between *LH8* and *OsMADS50*. Furthermore, no significant difference of the expression of *OsMADS50* was found between HJX74 and CSSL5. Therefore, whether *EH3* is allelic to *OsMADS50* remains to be investigated. Further fine mapping and molecular identification of *EH3* will be necessary to provide molecular evidence for the epistatic interaction between *LH8* and *EH3* in regulating heading date in rice.

## Methods

### Plant materials and growth conditions

CSSL5 is one of a set of 1,123 chromosome segment substitution lines, which were developed from backcross progenies (BC_6_F_2_) derived from a cross between an elite *indica* variety from south china, HJX74, as the recurrent parent and another *indica* variety Zihui100 as the donor parent.

Plants were grown under natural short-day (from July to November) and natural long-day (from March to July) conditions in a paddy field in Guangzhou (23°07′N, 113°15′E), China. Heading date was defined as the time when the first panicle emerged.

### High-throughput genotyping using whole-genome resequencing

DNA was extracted from 100 mg fresh rice leaves using the DNeasy Plant Mini Kit (QIAGEN Sciences). DNA was quantified using NanoDrop 1000 spectrophotometer (Thermo Scientific), and about 5 μg of DNA was used for preparation of libraries for Illumina sequencing according to the protocol for the Paired-End DNA Sample Prep kit (Illumina). The libraries were used for cluster generation on a flow cell and sequenced for 76 cycles on an Illumina Genome Analyzer IIx. Base calling and filtering of low-quality bases were done using sequence control software real-time analysis, Base calling (BCL) converter and the GERALD module (Illumina).

### DNA extraction and PCR amplification

Fresh leaves were collected at the seedling stage and then ground in liquid nitrogen. Microquantities of DNA were extracted from fresh leaves of each individual using a previously reported method^45^. Amplification was carried out on the program for the initial denaturing step with 94°C for 3 min, followed by 35 cycles for 30 s at 94°C, 30 s at 55°C, 30 s at 72°C, with a final extension at 72°C for 5 min. PCR products were separated on 6% non-denaturing polyacrylamide gel and detected using the silver staining method^46^.

### Linkage map and QTL analysis

We constructed the linkage map using Mapmaker/Exp3.0^47^. The Kosambi function was used to calculate genetic distances. QTL for heading date were estimated by Mapmaker/QTL 1.1^48^. Putative QTL were identified in regions exceeding 3.0 LOD (log-likelihood value).

### Evaluation of genetic interactions

To analyze genetic interactions between *LH8* and *EH3*, we classified a total of 359 and 225 F_2_ plants grown under NLD and NSD conditions in 2011, respectively, into nine classes according to the genotypes of SSR markers tightly linked to the QTLs (Id83-Id82 interval for *LH8*, and Id32-Id33 interval for *EH3*). We compared the mean DTH between the nine genotypes using two-way analysis of variance (ANOVA). Testing for each genetic interaction types (AA, AD, DA and DD) was done by orthogonal contrast test in STAR software (http://bbi.irri.org/).

### Mapping for *LH8*

F_2_ plants with homozygous *EH3* allele from HJX74 and heterozygous *LH8* allele from Zihui100 were selected to generate F_3_ population. A total of 559 and 1,600 F_3_ plants were grown to fine map *LH8* gene under NLD and NSD conditions in 2012, respectively. Each plant was genotyped using molecular markers and phenotyped by visual examination of the first panicle emergence. The F_4_ recombinant lines derived from the F_3_ population were used for progeny testing.

### RNA Extraction and QRT-PCR

Total RNA from 30d-old leaves were isolated using TRIZOL reagent (Invitrogen) following the manufacturer's instruction. First-strand cDNA was reverse transcribed from DNaseI-treated RNA with oligo-dT as the primer using ReverTra Ace kit (Toyobo). Gene expression was measured by QRT-PCR using the ABI 7500 system (Life technologies). The QRT-PCR was carried out in a total volume of 20 μl containing 1X SYBR Green Master Mix (Life technologies). We normalized the expression levels by using *UBQ5* gene as internal control. Each set of experiments was repeated three times, and the relative standard curve quantification method was used to evaluate quantitative variation. The QRT-PCR procedure was conducted at 94°C for 3 min, followed by 40 cycles at 94°C for 30 s, 58°C for 30 s, and 72°C for 30 s. The QRT-PCR primers were listed in Supplemental Table S3 online.

### Yeast two-hybrid assay

The cDNA fragments encoding the entire putative LH8 and Hd1 proteins were PCR-amplified, verified by sequencing and cloned into NdeI–EcoRI restriction sites of the MATCHMAKER two-hybrid prey vector pGADT7 or bait vector pGBKT7 (Clontech), respectively. The bait and prey constructs were co-transformed into the yeast strain AH109, and the transformed cells were plated on synthetic defined medium containing all essential amino acids except leucine and tryptophan (SD/-Leu/-Trp) medium and incubated at 30°C for 3 d and then diluted and applied onto SD/-Leu/-Trp/-His/-Ade medium and SD/-Leu/-Trp medium (as loading control) and cultured at 30°C for 2–3 days. The interaction between HYL1 and SE was used as a positive control^49^. The primers used in vector construction were listed in Supplemental Table S3 online.

## Author Contributions

Z.Q.L. and G.Q.Z. designed the experiments. Z.Q.L. wrote the manuscript. J.B.C. and M.Q. performed QTL analysis. J.B.C. and X.Y.L. performed map-based cloning. C.C. and H.T.Z. performed sequencing analysis. Y.H.W. performed real-time RT-PCR. R.Z.Z. and X.L.F. performed yeast-two-hybrid analysis. All authors commented on the manuscript.

## Supplementary Material

Supplementary Information Supplementary Table

## Figures and Tables

**Table 1:** Heading date QTLs detected in an F_2_ population from HJX74 x CSSL5 under NLD conditions

QTL	Chromosome	Marker interval	LOD^a^	PVE^b^	a^c^	d^d^
*EH3*	3	Id32-Id33	4.13	14.0%	−2.32	−3.24
*LH8*	8	Id83-Id82	53.12	65.3%	6.62	3.44

^a^Log-likelihood value.

^b^Percentage phenotypic variance explained by the QTL.

^c^Additive effect of the Zihui100 allele on days to heading.

^d^Dominant effect of the Zihui100 allele.

## References

[b1] Kobayashi Y. & Weigel D. Move on up, it's time for change--mobile signals controlling photoperiod-dependent flowering. Genes Dev 21, 2371–84 (2007). 17908925 10.1101/gad.1589007

[b2] Putterill J., Laurie R. & Macknight R. It's time to flower: the genetic control of flowering time. Bioessays 26, 363–73 (2004). 15057934 10.1002/bies.20021

[b3] Tsuji H., Taoka K. & Shimamoto K. Regulation of flowering in rice: two florigen genes, a complex gene network, and natural variation. Curr Opin Plant Biol 14, 45–52 (2011). 20864385 10.1016/j.pbi.2010.08.016

[b4] Yano M. *et al.* Hd1, a major photoperiod sensitivity quantitative trait locus in rice, is closely related to the *Arabidopsis* flowering time gene *CONSTANS*. Plant Cell 12, 2473–2484 (2000). 10.1105/tpc.12.12.2473 PMC102231 11148291

[b5] Hayama R., Yokoi S., Tamaki S., Yano M. & Shimamoto K. Adaptation of photoperiodic control pathways produces short-day flowering in rice. Nature 422, 719–22 (2003). 12700762 10.1038/nature01549

[b6] Doi K. *et al.* *Ehd1,* a B-type response regulator in rice, confers short-day promotion of flowering and controls *FT*-like gene expression independently of *Hd1*. Genes Dev 18, 926–36 (2004). 15078816 10.1101/gad.1189604 PMC395851

[b7] Komiya R., Yokoi S. & Shimamoto K. A gene network for long-day flowering activates *RFT1* encoding a mobile flowering signal in rice. Development 136, 3443–50 (2009). 19762423 10.1242/dev.040170

[b8] Izawa T. Adaptation of flowering-time by natural and artificial selection in *Arabidopsis* and rice. J Exp Bot 58, 3091–7 (2007). 17693414 10.1093/jxb/erm159

[b9] Izawa T., Takahashi Y. & Yano M. Comparative biology comes into bloom: genomic and genetic comparison of flowering pathways in rice and *Arabidopsis*. Curr Opin Plant Biol 6, 113–20 (2003). 12667866 10.1016/s1369-5266(03)00014-1

[b10] Komiya R., Ikegami A., Tamaki S., Yokoi S. & Shimamoto K. *Hd3a* and *RFT1* are essential for flowering in rice. Development 135, 767–74 (2008). 18223202 10.1242/dev.008631

[b11] Wu C. *et al.* *RID1*, encoding a Cys2/His2-type zinc finger transcription factor, acts as a master switch from vegetative to floral development in rice. Proc Natl Acad Sci USA 105, 12915–20 (2008). 18725639 10.1073/pnas.0806019105 PMC2529042

[b12] Park S. J. *et al.* *Rice Indeterminate 1* (*OsId1*) is necessary for the expression of *Ehd1* (*Early heading date 1*) regardless of photoperiod. Plant J 56, 1018–29 (2008). 18774969 10.1111/j.1365-313X.2008.03667.x

[b13] Matsubara K. *et al.* *Ehd2*, a rice ortholog of the maize *INDETERMINATE1* gene, promotes flowering by up-regulating *Ehd1*. Plant Physiol 148, 1425–35 (2008). 18790997 10.1104/pp.108.125542 PMC2577255

[b14] Matsubara K. *et al.* *Ehd3*, encoding a plant homeodomain finger-containing protein, is a critical promoter of rice flowering. Plant J 66, 603–12 (2011). 21284756 10.1111/j.1365-313X.2011.04517.x

[b15] Gao H. *et al.* *Ehd4* encodes a novel and Oryza-genus-specific regulator of photoperiodic flowering in rice. PLoS Genet 9, e1003281 (2013). 23437005 10.1371/journal.pgen.1003281 PMC3578780

[b16] Kim S. L., Lee S., Kim H. J., Nam H. G. & An G. *OsMADS51* is a short-day flowering promoter that functions upstream of *Ehd1*, *OsMADS14*, and *Hd3a*. Plant Physiol 145, 1484–94 (2007). 17951465 10.1104/pp.107.103291 PMC2151696

[b17] Lee S., Kim J., Han J. J., Han M. J. & An G. Functional analyses of the flowering time gene *OsMADS50*, the putative *SUPPRESSOR OF OVEREXPRESSION OF CO 1/AGAMOUS-LIKE 20* (*SOC1*/*AGL20*) ortholog in rice. Plant J 38, 754–64 (2004). 15144377 10.1111/j.1365-313X.2004.02082.x

[b18] Ryu C. H. *et al.* *OsMADS50* and *OsMADS56* function antagonistically in regulating long day (LD)-dependent flowering in rice. Plant Cell Environ 32, 1412–27 (2009). 19558411 10.1111/j.1365-3040.2009.02008.x

[b19] Bian X. F. *et al.* Heading date gene, *dth3* controlled late flowering in O. Glaberrima Steud. by down-regulating *Ehd1*. Plant Cell Rep 30, 2243–54 (2011). 21830130 10.1007/s00299-011-1129-4

[b20] Xue W. *et al.* Natural variation in *Ghd7* is an important regulator of heading date and yield potential in rice. Nat Genet 40, 761–7 (2008). 18454147 10.1038/ng.143

[b21] Wei X. *et al.* *DTH8* suppresses flowering in rice, influencing plant height and yield potential simultaneously. Plant Physiol 153, 1747–58 (2010). 20566706 10.1104/pp.110.156943 PMC2923886

[b22] Yan W. H. *et al.* A major QTL, *Ghd8*, plays pleiotropic roles in regulating grain productivity, plant height, and heading date in rice. Mol Plant 4, 319–30 (2011). 21148627 10.1093/mp/ssq070

[b23] Dai X. *et al.* *LHD1*, an allele of *DTH8*/*Ghd8*, controls late heading date in common wild rice (*Oryza rufipogon*). J Integr Plant Biol 54, 790–9 (2012). 22963226 10.1111/j.1744-7909.2012.01166.x

[b24] Andres F., Galbraith D. W., Talon M. & Domingo C. Analysis of *PHOTOPERIOD SENSITIVITY5* sheds light on the role of phytochromes in photoperiodic flowering in rice. Plant Physiol 151, 681–90 (2009). 19675157 10.1104/pp.109.139097 PMC2754645

[b25] Lee Y. S. *et al.* *OsCOL4* is a constitutive flowering repressor upstream of *Ehd1* and downstream of *OsphyB*. Plant J 63, 18–30 (2010). 20409004 10.1111/j.1365-313X.2010.04226.x

[b26] Peng L. T., Shi Z. Y., Li L., Shen G. Z. & Zhang J. L. Ectopic expression of *OsLFL1* in rice represses *Ehd1* by binding on its promoter. Biochem Biophys Res Commun 360, 251–6 (2007). 17592727 10.1016/j.bbrc.2007.06.041

[b27] Peng L. T., Shi Z. Y., Li L., Shen G. Z. & Zhang J. L. Overexpression of transcription factor *OsLFL1* delays flowering time in *Oryza sativa*. J Plant Physiol 165, 876–85 (2008). 17913295 10.1016/j.jplph.2007.07.010

[b28] Fukuoka S., Ebana K., Yamamoto T. & Yano M. Integration of Genomics into Rice Breeding. Rice 3, 131–137 (2010).

[b29] Yamamoto T., Yonemaru J. & Yano M. Towards the understanding of complex traits in rice: substantially or superficially? DNA Res 16, 141–54 (2009). 19359285 10.1093/dnares/dsp006 PMC2695773

[b30] Fukuoka S., Nonoue Y. & Yano M. Germplasm enhancement by developing advanced plant materials from diverse rice accessions. Breeding Sci 60, 509–517 (2010).

[b31] Zhang G. Q. *et al.* The construction of a library of single segment substitution lines in rice (*Oryza sativa* L.). Rice Genetics Newsletter 21, 85–87 (2004).

[b32] Xi Z. Y. *et al.* Development of a wide population of chromosome single-segment substitution lines in the genetic background of an elite cultivar of rice (*Oryza sativa* L.). Genome 49, 476–84 (2006). 16767172 10.1139/g06-005

[b33] Yu S. B. *et al.* Identification of quantitative trait loci and epistatic interactions for plant height and heading date in rice. Theor Appl Genet 104, 619–625 (2002). 12582666 10.1007/s00122-001-0772-5

[b34] Lin H. X., Liang Z. W., Sasaki T. & Yano M. Fine mapping and characterization of quantitative trait loci *Hd4* and *Hd5* controlling heading date in rice. Breeding Sci 53, 51–59 (2003).

[b35] Gu X. Y. & Foley M. E. Epistatic interactions of three loci regulate flowering time under short and long daylengths in a backcross population of rice. Theor Appl Genet 114, 745–54 (2007). 17171390 10.1007/s00122-006-0475-z

[b36] Uwatoko N. *et al.* Epistasis among the three major flowering time genes in rice: coordinate changes of photoperiod sensitivity, basic vegetative growth and optimum photoperiod. Euphytica 163, 167–175 (2008).

[b37] Liu S. *et al.* Genetic analysis and fine mapping of *LH1* and *LH2*, a set of complementary genes controlling late heading in rice (*Oryza sativa* L.). Breeding Sci 62, 310–319 (2012). 10.1270/jsbbs.62.310 PMC3528327 23341744

[b38] Maity S. N. & de Crombrugghe B. Role of the CCAAT-binding protein CBF/NF-Y in transcription. Trends Biochem Sci 23, 174–8 (1998). 9612081 10.1016/s0968-0004(98)01201-8

[b39] Mantovani R. The molecular biology of the CCAAT-binding factor NF-Y. Gene 239, 15–27 (1999). 10571030 10.1016/s0378-1119(99)00368-6

[b40] Goda H. *et al.* Nuclear translocation of the heterotrimeric CCAAT binding factor of *Aspergillus oryzae* is dependent on two redundant localising signals in a single subunit. Arch Microbiol 184, 93–100 (2005). 16163515 10.1007/s00203-005-0014-3

[b41] Tuncher A., Sprote P., Gehrke A. & Brakhage A. A. The CCAAT-binding complex of eukaryotes: evolution of a second NLS in the HapB subunit of the filamentous fungus *Aspergillus nidulans* despite functional conservation at the molecular level between yeast, *A. nidulans* and human. J Mol Biol 352, 517–33 (2005). 16098534 10.1016/j.jmb.2005.06.068

[b42] Wenkel S. *et al.* *CONSTANS* and the CCAAT box binding complex share a functionally important domain and interact to regulate flowering of *Arabidopsis*. Plant Cell 18, 2971–84 (2006). 17138697 10.1105/tpc.106.043299 PMC1693937

[b43] Takano M. *et al.* Distinct and cooperative functions of *phytochromes A*, *B*, and *C* in the control of deetiolation and flowering in rice. Plant Cell 17, 3311–25 (2005). 16278346 10.1105/tpc.105.035899 PMC1315371

[b44] Li D. *et al.* Functional characterization of rice *OsDof12*. Planta 229, 1159–69 (2009). 19198875 10.1007/s00425-009-0893-7

[b45] Shan J. X., Zhu M. Z., Shi M., Gao J. P. & Lin H. X. Fine mapping and candidate gene analysis of *spd6*, responsible for small panicle and dwarfness in wild rice (*Oryza rufipogon* Griff.). Theor Appl Genet 119, 827–36 (2009). 19588119 10.1007/s00122-009-1092-4

[b46] Li W. T., Zeng R. Z., Zhang Z. M. & Zhang G. Q. Mapping of *S-b* locus for F1 pollen sterility in cultivated rice using PCR based markers. Acta Botanica Sinica 44, 463–467 (2002).

[b47] Lander E. S. *et al.* MAPMAKER: an interactive computer package for constructing primary genetic linkage maps of experimental and natural populations. Genomics 1, 174–81 (1987). 3692487 10.1016/0888-7543(87)90010-3

[b48] Lincoln S., Daly M. & Lander E. Mapping genes controlling quantitative traits with MAPMAKER/QTL1.1. 2nd edition (Whitehead Institute Technical Report, Cambridge, Massachusetts, 1992).

[b49] Yang L., Liu Z., Lu F., Dong A. & Huang H. SERRATE is a novel nuclear regulator in primary microRNA processing in *Arabidopsis*. Plant J 47, 841–50 (2006). 10.1111/j.1365-313X.2006.02835.x 16889646

