# Supplementary material for: Characterization of Epistatic Interaction of QTLs LH8 and EH3 Controlling Heading Date in Rice
Source: Sci Rep. 2014 Mar 3;4:4263. doi: 10.1038/srep04263 (PMC3939452; doi:10.1038/srep04263)
Supplement: Supplementary Information — Supplementary Table [file srep04263-s1.doc]

**Characterization of Epistatic Interaction of QTLs *LH8* and *EH3* Controlling Heading Date in Rice**

Jingbin Chen^1, 2^, Xiaoyan Li^1, 3^, Cheng Cheng^1^, Yahuan Wang^1^, Mao Qin^1^, Haitao Zhu^1^, Ruizhen Zeng^1^, Xuelin Fu^1^, Ziqiang Liu^1*^ & Guiquan Zhang^1*^

Table S1. Distribution of the substituted segments of W05-01-11-02-07-06

| Segment | Chromosome | Start position | End position | Length (bp) |
| --- | --- | --- | --- | --- |
| 1 | 1 | 12,280,590 | 18,520,953 | 6,240,364 |
| 2 | 3 | 1 | 10,298,783 | 10,298,783 |
| 3 | 3 | 29,227,946 | 29,234,792 | 6,847 |
| 4 | 3 | 29,303,386 | 29,575,811 | 272,426 |
| 5 | 7 | 27,787,461 | 28,390,030 | 602,570 |
| 6 | 8 | 3,561,924 | 4,557,660 | 995,737 |
| 7 | 9 | 19,852,753 | 19,865,682 | 12,930 |
| 8 | 9 | 20,832,167 | 21,363,098 | 530,932 |
| 9 | 9 | 21,385,619 | 21,810,525 | 424,907 |
| 10 | 12 | 19,932,802 | 20,793,050 | 860,249 |
| 11 | 12 | 27,134,066 | 27,531,856 | 397,791 |
| Total |  |  |  | 20,643,536 |

Table S2. Trait measurements of HJX74, W05-01-11-02-07-06 and NIL-*LH8* under NSD conditions.

| Trait | HJX74 | W05-01-11-02-07-06 | NIL-*LH8* |
| --- | --- | --- | --- |
| Plant height (cm) | 89.23±0.92 a | 94.57±1.11 b | 93.13±0.52 b |
| No. of effective panicles | 9.00±0.32 ab | 10.00±0.45 b | 8.00±0.55 a |
| No. of spikelets per panicle | 161.98±8.09 a | 228.59±4.15 b | 206.71±11.35 b |
| Setting rate (%) | 85.35±2.00 b | 78.53±1.34 a | 73.94±1.48 a |
| 1000-grain weight (g) | 11.56±0.16 b | 9.96±0.06 a | 10.25±0.13 a |

Note: Each data represents mean ± s.d. (n = 25). Lowercase letters after data (a, b, ab) indicate the result of Duncan’s multiple comparison at the 0.05 significance level.

Table S3. Primers used in this study.

| Primer name | Sequence 5'-3' |
| --- | --- |
| For linkage analysis and mapping | |
| Id83F | AAACTCATCAGCTGGACTTGG |
| Id83R | TTTGCAGCCCATTTAATTTGT |
| IND112F | TTTTCTCAATGCACAAGGAGG |
| IND112R | AAAAGCCAATGTTGAAAAAGAAA |
| RM22475F | ACCTCCTGCAGCTGGTCCTATACC |
| RM22475R | CTGCTGTTCTTGGTGGTGATGG |
| Id85F | ATGGATCAATTCCGCTTTCTT |
| Id85R | TACAGTGATGTGGCCTTGACA |
| Id87F | CAAAGTTCGACACGGGATAAA |

Table S3. Continued.

| Primer name | Sequence 5'-3' |
| --- | --- |
| Id87R | ACTTGTCGAGCTGCGGTTAT |
| Id811F | AACTCCAAATGGCTCGTCTTT |
| Id811R | CTCTGCGGATTTCATGGTAGA |
| RM25F | GGAAAGAATGATCTTTTCATGG |
| RM25R | CTACCATCAAAACCAATGTTC |
| Id118F | AGGGCATGTTCAGATTGTAGC |
| Id118R | GCGTGTTGGTGAATAACCACT |
| Id82F | TTTGGCAGAGAGAGAGAGAGAGA |
| Id82R | GGATTATCCGATATTTTGATCG |
| Id32F | CCGTTGCTCTTCTCTCTCCTT |
| Id32R | TGACAGGTGGGACCATGTATT |
| Id33F | GCTCGAGCCTAGGCAGATAAG |
| Id33R | GGTCATCTGACCATAACATGGA |
|  |  |
| For real-time RT-PCR | |
| qLH8-F | CAGGAGTGCGTGTCGGAGTT |
| qLH8-R | GGTCGTCGCCGTTGATGGT |
| qHd1-F | TCAGCAACAGCATATCTTTCTCATCA |
| qHd1-R | TCTGGAATTTGGCATATCTATCACC |
| qOsMADS50-F | CAGGCCAGGAATAAGCTGGAT |
| qOsMADS50-R | TTAGGATGGTTTGGTGTCATTGC |
| qEhd1-F | GGATGCAAGGAAATCATGGA |
| qEhd1-R | AATCCCATCGGAAATCTTGG |
| qHd3a-F | GCTCACTATCATCATCCAGCATG |
| qHd3a-R | CCTTGCTCAGCTATTTAATTGCATAA |
| qRFT1-F | GTCGCCACCGTCTACTTCAACT |
| qRFT1-R | TACAGCTAGGCAGGTCTCAGCT |
| qUBQ5-F | ACCACTTCGACCGCCACTACT |
| qUBQ5-R | ACGCCTAAGCCTGCTGGTT |
|  |  |
| For Y2H vectors |  |
| LH8-F1-NdeI | AcatatgAAGAGTAGGAAGAGCTA |
| LH8-R1-EcoRI | AgaattcCTAATTAGTACTCCTCTTTAGT |
| LH8-R2-EcoRI | AgaattcTCATGCGTGGAGCCAGAG |
| Hd1-F1-NdeI | AcatatgATGAATTATAATTTTGGTGGC |
| Hd1-R1- EcoRI | CgaattcTTATTCATAACACAGATTGTCTAGA |
| Hd1-R2- EcoRI | AgaattcTCAGAACCATGGAACAGTA |

Note: F, forward primer; R, reverse primer. Underlines indicate the restriction sites.
